# Association of Physical Activity Intensity with All-Cause Mortality in Cancer Survivors: A National Prospective Cohort Study

**DOI:** 10.3390/cancers14235760

**Published:** 2022-11-23

**Authors:** Qiguang Li, Xueqiang Pan, Xiao Li, Wei Huang

**Affiliations:** 1 Department of Oncology, Shandong Provincial Hospital Affiliated to Shandong First Medical University, Jinan 250021, China; 2 Department of Oncology, Xiangya Hospital, Central South University, Changsha 410017, China; 3 Research Center of Carcinogenesis and Targeted Therapy, Xiangya Hospital, Central South University, Changsha 410017, China; 4 National Clinical Research Center of Geriatric Disorders, Xiangya Hospital, Changsha 410017, China

**Keywords:** physical activity, all-cause mortality, NHIS, cancer survivors

## Abstract

**Simple Summary:**

Insufficient physical activity (PA) is a global health issue with significant disease burden. Increasing evidence suggests that higher PA levels have protective effects against chronic diseases. Few prospective cohort studies have explored the association between PA levels and mortality in cancer survivors. Using a large nationally representative survey population of United States adults, this study demonstrated a beneficial association between leisure-time PA and all-cause mortality in cancer survivors. There was a nonlinear relationship between the total PA and the risk of all-cause mortality. Threshold effect analysis demonstrated that cancer survivors should perform PA for at least 1 h per week and performing more PA could provide additional survival benefits for cancer survivors.

**Abstract:**

We designed this study to investigate the associations between physical activity (PA) and the risk of all-cause mortality in cancer survivors using a nationally representative cohort of US adults. This cohort study included 13 cycles of the National Health Interview Surveys, and by matching participants with the National Death Index (2015), survival status was determined. The main outcome was all-cause mortality during follow-up. A total of 20,088 participants aged 62.2 (15.9) years (62.4% women) were analyzed. After an average follow-up of 117.5 months, 7214 (35.9%) participants died. Compared with inactive cancer survivors, we observed a 25% lower all-cause mortality risk among participants performing PA 10 min to 1 h/week (hazard ratio [HR] = 0.75, 95% confidence interval [CI] = 0.67–0.85), a 28% lower risk among those performing PA 1–2.5 h/week (HR = 0.72, 95% CI = 0.67–0.78), a 34% lower risk among those performing PA 2.5–5 h/week (HR = 0.66, 95% CI = 0.60–0.72), a 37% lower risk among those performing PA 5–7.5 h/week (HR = 0.63, 95% CI = 0.56–0.70), a 47% lower risk among those performing PA 7.5–13.3 h/week (HR = 0.53, 95% CI = 0.47–0.61), and a 43% lower risk among those performing PA 13.3–24 h/week (adjusted HR = 0.53, 95% CI = 0.49–0.66). In cancer survivors, leisure-time PA was associated with a lower all-cause mortality. Inactive cancer survivors should be encouraged to perform more PA to reduce the risk of all-cause mortality.

## 1. Introduction

Insufficient physical activity (PA) is a global health issue, with a significant disease burden. Previous epidemiologic studies have provided scientific evidence that regular PA is frequently associated with a decreased risk of mortality [1,2,3,4]. In 2008, the United States (US) guidelines on PA recommended that adults should take part in a minimum of 150 min/week of moderate PA or 75 min/week of vigorous PA, or an equivalent combination of both [5]. Increasing evidence suggests that higher PA levels have a protective effect against chronic diseases by preventing multiple chronic disorders (such as type 2 diabetes, malignancy, and cardiovascular disease) and lowering mortality [6,7]. Therefore, participation in PA is essential because it lowers the mortality rates of chronic diseases and correspondingly lowers healthcare expenditures and productivity losses. However, the majority of previous studies have only focused on the link between PA and mortality among the general population or patients with cardiovascular disease [1,2,3,4,8]. In a cohort study of a nationally representative sample of US cancer survivors, the combination of daily sitting time and leisure time PA was associated with the highest risks of death from all causes and cancer [9]. A meta-analysis of 136 studies showed that recreational PA improved survival outcomes in all cancers combined [10]. According to US recommendations, women with breast cancer may live longer if they follow PA guidelines [11]. Patients with cancer who are habitually active have a 39% lower risk of all-cause mortality than those who are habitually inactive and a 36% lower risk of cancer-specific mortality than those who are habitually inactive [12]. However, few prospective cohort studies have explored the nonlinear association between PA levels and mortality in cancer survivors. Furthermore, these studies that explored the association between PA levels and mortality risk among adults often excluded cancer survivors at baseline [3,4,13]. Most individuals diagnosed with cancer in the US survive for ≥ 5 years, resulting in a growing population of cancer survivors [14,15]. Individuals with a history of cancer are at a higher risk of mortality and morbidity, and are often less physically active than those without a history of cancer. Thus, this cohort requires specific attention to reduce the risk of premature death. However, there is little research on how PA affects cancer survivors’ survival and the dose–response relationship. Using a large nationally representative sample of the US population, the present study examined the relationship between PA and the risk of mortality among cancer survivors.

## 2. Materials and Methods

### 2.1. Study Design and Population

We analyzed nationally representative data collected from 386,887 US individuals aged ≥ 18 years who participated in the 1997–2009 National Health Interview Survey (NHIS). It is a national yearly cross-sectional household interview survey that collects details on a range of self-reported health information for the civilian, noninstitutionalized US population. It was conducted in 1957 using a face-to-face interview format and aimed to supervise the health conditions of the US population by the Centers for Disease Control and Prevention. The NHIS adopts a stratified multistage sample design to implement representative sampling of the target population. The specific method of the NHIS sampling design has been described previously [16]. NHIS 1997–2009 was used in this study to ensure consistency in self-reported responses of the survey participants because the NHIS database was revised in 1997. As the NHIS database was deidentified and made publicly available, this study did not require institutional review board approval.

**Figure 1:**
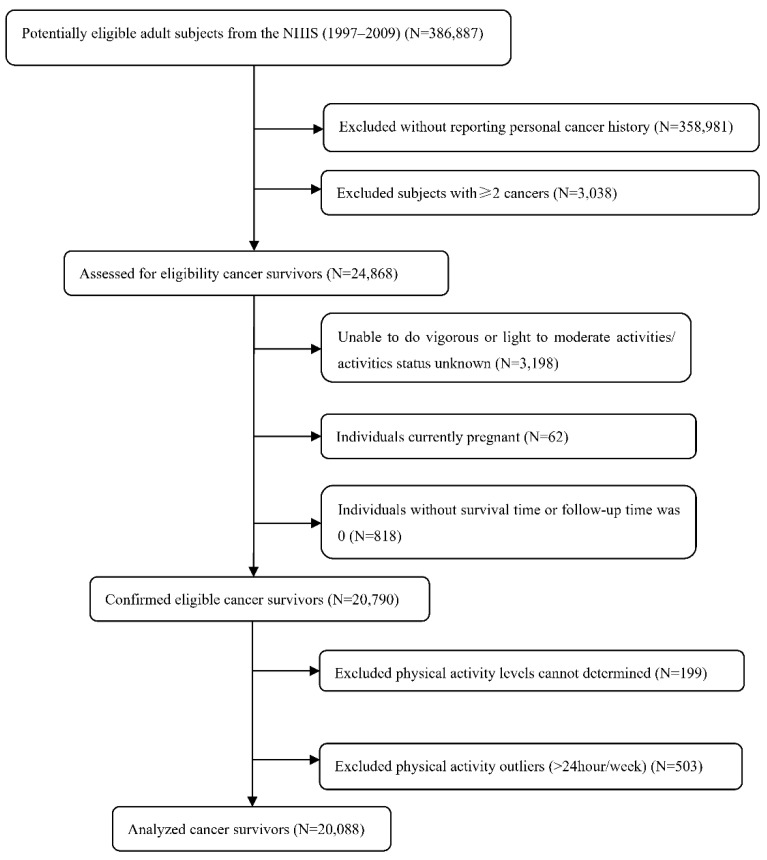
Flowchart of eligible and ineligible cancer survivors.

Based on each individual’s identification code, the data from the NHIS baseline were merged with the National Death Index (NDI) data up to 31 December 2015. Among the 386,887 participants from 13 cross-sectional cycles performed between 1997 and 2009, 358,981 individuals were excluded because they did not report a personal cancer history. Subjects with more than two cancers were excluded (*n* = 3038). A total of 3198 participants who were unable to perform vigorous or light-to-moderate activities or whose activity status were unknown were excluded. A total of 62 individuals who were currently pregnant and 818 individuals without a survival time or follow-up time of 0 were excluded. Finally, we excluded those whose PA levels could not be determined (*n* = 199). A total of 503 individuals with PA > 24 h/week as outliers were removed (Figure S1). Thus, 20,088 adult cancer survivors were included in this study (Figure 1).

### 2.2. Study Outcome

The main endpoint was all-cause mortality during follow-up. The subjects in NHIS with age ≥ 18 years were included for survival follow-up. To explore the survival status of NHIS individuals, we utilized a probabilistic record-matching approach with NDI documents. It has been confirmed that the NDI archives-matching approach presents perfect performance. The correct categorization of deceased individuals was 96.1%, whereas the correct categorization of non-deceased individuals was 99.4% [17]. The details of death were defined based on ICD-10 revision codes. All-cause mortality was the primary outcome measure. The secondary endpoints were cardiovascular disease mortality and cancer-specific mortality.

### 2.3. Study Exposure

First, cancer survivors were identified using the following survey questionnaire: “Have you ever been told by a doctor or other health professional that you had cancer or a malignancy of any kind?” Subsequently, survey respondents who answered yes to this question were deemed cancer survivors in this study. The main study exposure was PA intensity, measured by the frequency and duration of PA for the included individuals. Leisure-time PA was measured using self-report questionnaires. Two survey questionnaires investigating the frequency and duration of PA were used to measure the total PA. Vigorous PA was determined according to the following questionnaires: “About how long do you do these vigorous activities each time?” and “How often do you do vigorous activities for at least 20 min that cause heavy sweating or large increases in breathing or heart rate?” The following questionnaire was used to define light or moderate PA: “About how long do you do these light or moderate activities each time?” and “How often do you do light or moderate activities for at least 20 min that cause only light sweating or a slight-to-moderate increase in breathing or heart rate?” All study individuals were asked to complete questionnaires relating to the frequency (times/week) and duration (hours/week) of their PA. Frequency was measured as PA lasting for more than 10 min and categorized into vigorous PA (such as competitive sports, running, playing squash, and faster cycling) and light or moderate PA (such as gardening and dancing). To quantify the total PA, we multiplied the frequency by the duration of activity to yield the PA in min/week. Recent PA guidelines and studies recommend 1 min of vigorous PA equivalent to 2 min of moderate PA [2,18]. By converting vigorous PA into its moderate PA equivalent, we were able to calculate the total PA level (h/week) for each subject. Based on previous studies, total leisure time PA was grouped into seven subgroups: 0, 10 min–1 h, 1–2.5 h, 2.5–5 h, 5–7.5 h, 7.5–13.3 h, and 13.3–24 h/week [1,2]. Individuals who performed no (or zero) PA served as the reference group.

### 2.4. Study Covariates

In this study, information about the participants’ demographic characteristics, lifestyle, and clinical risk factors was obtained as covariates. Demographic characteristics included age, sex, ethnicity or race (white, black, and others), and marital status (married/living with partner, divorced/separated/widowed, and never married). Lifestyle included body mass index (BMI), alcohol drinking status (lifetime abstainer, former drinker, current drinker), PA, and smoking status (never, former, current, and smoker but current status unknown). A person’s BMI was calculated by dividing their weight in kilograms by their height squared and categorizing them as underweight (<18.5 kg/m^2^), normal weight (18.5–24.9 kg/m^2^), overweight (25–29.9 kg/m^2^), and obese (≥30 kg/m^2^). Using self-reported yes/no answers to two successive survey questionnaires, we identified the smoking history of individuals: (1) Have you smoked at least 100 cigarettes in your entire life? (2) Do you still smoke cigarettes? Based on the responses to these questions, we categorized the participants as never smokers, former smokers, and current smokers. Several other risk factors were evaluated, including self-reported diagnosis of coronary heart disease, hypertension, stroke, asthma, diabetes, and angina.

### 2.5. Statistical Analysis

Differences in the distribution of the baseline characteristics of individuals were expressed as percentages for categorical variables. Multivariable Cox proportional hazards regression was used to evaluate the risk of all-cause, cardiovascular disease (CVD), and cancer mortality with total PA and to calculate the hazard ratios (HRs) and 95% confidence intervals (CIs), while adjusting for multiple potential confounding factors. The proportional hazard assumption was explored based on Schoenfeld residual plots, and no obvious violations were found. The survival time (transformed to years) was calculated as the day from the household interview to death or the censor point. Kaplan–Meier curves were plotted for all-cause, CVD, and cancer-specific mortality according to PA status (with vs. without). Using sequential adjustments for confounding covariates, three models were developed to determine the influence of different possible confounding factors on PA and mortality. Model 1 (unadjusted model) was the baseline model without adjustment for potential confounders. Model 2 was adjusted for age, race, sex, smoking status, BMI, marital status, and alcohol consumption. Model 3 was additionally adjusted for a history of hypertension, coronary heart disease, asthma, angina, myocardial infarction, diabetes, and stroke. The dose–response relationship between PA and mortality risk was further investigated using Cox models with restricted cubic splines to account for potential nonlinearity. Furthermore, to calculate the inflection point, we used a two-piecewise Cox regression algorithm if the evidence indicated nonlinearity. Subgroup analysis stratified by age (<40, 40–59, and ≥60 years), sex, BMI (<18.5, 18.5–24.9, 25–29.9, and ≥30), race/ethnicity (white, black, and other), marital status, alcohol drinking, smoking status, diabetes, hypertension, coronary heart disease, angina, myocardial infarction, stroke, and asthma was used to evaluate whether the link between PA and all-cause mortality changed among these variables. We conducted tests for linear trends by entering each PA subgroup as a continuous variable in the models. To investigate the statistical significance of interaction effects, we constructed interaction terms between seven PA subgroups (as a continuous variable) and the exposures of multiple variables. A Wald test was used for dichotomous variables, and a likelihood ratio test was used for multilevel variables.

Multiple sensitivity analyses were performed to verify the robustness of the primary outcomes. First, to exclude possible reverse causality, we performed a sensitivity analysis by removing cancer survivors who died within the first 24 months of follow up. Second, we excluded participants with any skin cancer during PA measurement. Third, observational epidemiological studies often encounter unmeasured confounding factors. Thus, an e-value algorithm was used to quantify the minimum strength of the association needed to fully explain the observed associations between PA and unmeasured predictors [19]. Statistical analysis was performed using R software version 3.6.3 (R Statistical Software, R Foundation for Statistical Computing, Vienna, Australia) and STATA statistical software (Stata Corporation, College Station, TX, USA, version 16.0). A two-sided *p* value less than 0.05 was considered statistically significant.

## 3. Results

### 3.1. Population Characteristics

From 386,887 consecutive individuals that participated in NHIS 1997–2009, the study population finally included a total of 20,088 US cancer survivors (7548 men and 12,540 women) aged 18–85 years at baseline. The number of cancer survivors in each cycle is illustrated in Figure S2. Among all cancer survivors, breast cancer, skin non melanoma, prostate cancer, and cervix cancer rank as the top four (Figure S3). Table 1 illustrates the basic clinical characteristics of the included populations and between the seven groups of the PA levels. Survivors of cancer who performed the most PA were usually younger, less likely to drink alcohol, married, with a lower body mass index, less likely to smoke, and with fewer co-morbidities.

### 3.2. The Relationship between PA and Cancer Survivors’ Mortality

**Figure 2:**
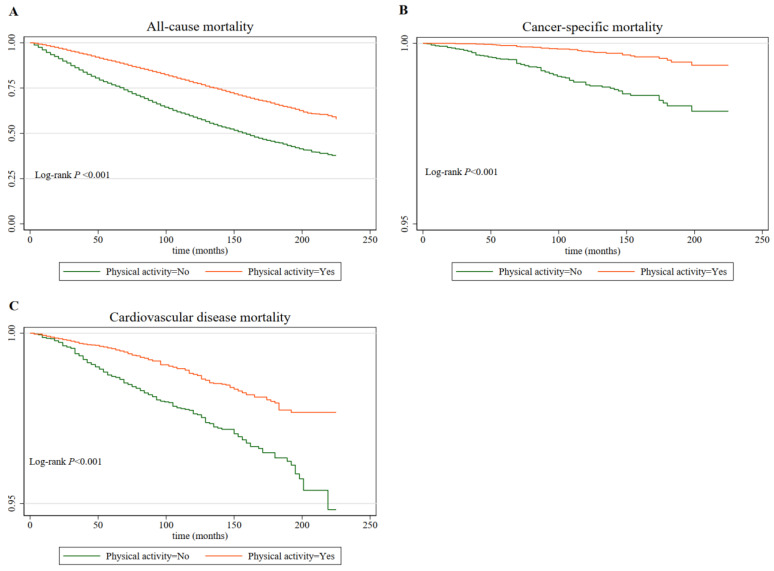
Kaplan–Meier curves for all-cause mortality (**A**), cancer mortality (**B**), and cardio vascular disease mortality (**C**) by physical activity levels (with vs. without).

During a follow-up period of 9.8 years, 7214 (35.9%), cancer survivors died from all causes (347 CVD deaths and 115 cancer-specific deaths). In unadjusted Kaplan–Meier analyses, the risks of all-cause mortality (Figure 2A), cancer-specific mortality (Figure 2B), and CVD mortality (Figure 2C) were higher among cancer survivors who were performed no PA (all *p* < 0.001).

**Figure 3:**
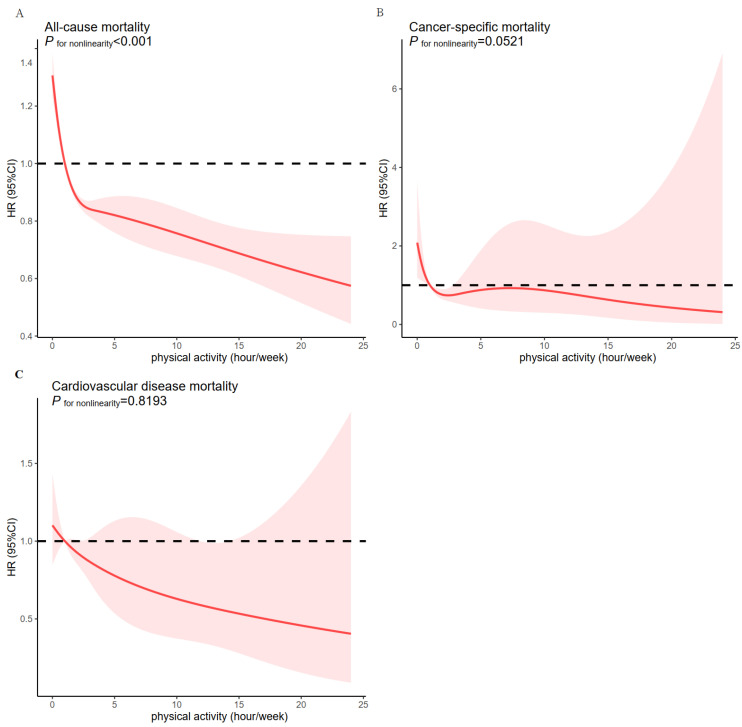
Association between physical activity and mortality by smooth curve fitting. All-cause mortality (**A**), cancer mortality (**B**), and cardiovascular disease mortality (**C**). Adjustment for age, sex, body mass index, race, marital status, alcohol drinking, smoking status, coronary heart disease, hypertension, stroke, asthma, diabetes, angina, and myocardial infarction. The red line demonstrates the risk of mortality, and the red ribbons illustrate its 95% confidence interval.

First, we treated PA as a continuous variable and calculated the HRs per h increment in PA intensity. It was revealed that for every 1 h increase in PA, the relative risk of mortality decreased by 8% in the nonadjusted model (model 1), 6% in model 2, and 5% in the fully adjusted model (Table 2). When inactive cancer survivors were used as reference, other levels of PA were associated with a lower risk of all-cause mortality (Table 2). Specifically, when adjusted for multiple confounding, cancer survivors performing 10 min–1 h, 1–2.5 h, 2.5–5 h, 5–7.5 h, 7.5–13.3 h, and 13.3–24 h of PA experienced a 25% (HR = 0.75, 95% CI = 0.67–0.85), 28% (HR = 0.72, 95% CI = 0.67–0.78), 34% (HR = 0.66, 95% CI = 0.60–0.72), 37% (HR = 0.63, 95% CI = 0.56–0.70), 47% (HR = 0.53, 95% CI = 0.47–0.61), and 43% (HR = 0.57, 95% CI = 0.49–0.66) lower risk of all-cause mortality, respectively (*p* for trend < 0.001). Cancer (*p* = 0.011) and CVD mortalities (*p* = 0.005) were inversely associated with increasing PA after adjustment for multiple covariates. Cox models with penalized splines presented a nonlinear association between the total amount of PA (continuous variable) and all-cause mortality (*p* for nonlinearity < 0.001, Figure 3A). The threshold effect analysis demonstrated that the turning point of PA was located at 1 h/week. Every 1 h/week increase in PA demonstrated a 30% decrease in the probability of all-cause mortality (HR = 0.70, 95% CI = 0.66–0.75), when the turning point was less than 1 h/week, and a 2% decrease (HR = 0.98, 95% CI = 0.97–0.99) on the right side of the turning point (Table 3). However, the nonlinear relationship was not apparent in cancer-specific mortality (Figure 3B) or CVD-specific mortality (Figure 3C).

### 3.3. Subgroup Analyses

Significant interactions of covariates (smoking status, hypertension, and coronary heart disease) with levels of PA were found on all-cause mortality (all *p* < 0.05), which implies that the overall findings differ in these strata. In subgroup analyses, there was a particularly strong association between PA and all-cause mortality among survivors with the following characteristics: older cancer survivors (≥60 years), men and women, overweight (25–29.9 kg/m^2^) and obese (≥30 kg/m^2^), white race, married/living with partner, divorced/separated/widowed, lifetime abstainer, former drinker, current drinker, without diabetes, with hypertension and without hypertension, without coronary heart disease, without angina, with and without myocardial infarction, stroke, former smoking, current smoking, and asthma (Table 4).

### 3.4. Sensitivity Analyses

**Figure 4:**
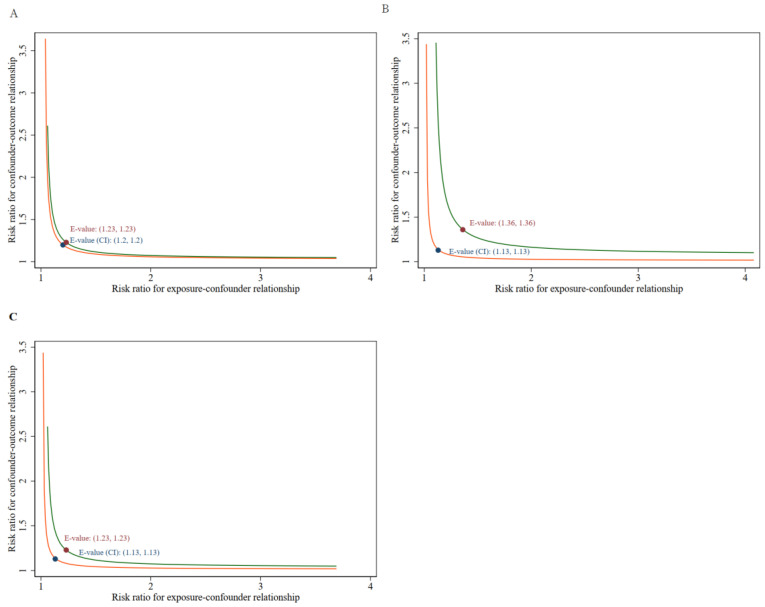
E-value together with the lower 95% confidence intervals and point estimate in (**A**) all-cause mortality, (**B**) cancer-specific, and (**C**) cardiovascular mortality.

Several sensitivity analyses were conducted to validate the main findings. First, excluding 1846 patients with skin cancer at baseline had little effect on all-cause mortality. As demonstrated in Table S1, when PA was treated as a continuous variable, we found that for every 1 h increase in PA, the relative risk of mortality decreased by 8% in model 1, 5% in model 2, and 5% in the fully adjusted model. These findings were robust when PA was used as a categorical variable. In the fully adjusted model, compared to individuals with physical inactivity at baseline, the level of PA was related to a strikingly decreased risk of all-cause mortality. Participants performing 10 min–1 h, 1–2.5 h, 2.5–5 h, 5–7.5 h, 7.5–13.3 h, and 13.3–24 h of PA were found to have a 27% (HR = 0.73, 95% CI = 0.63–0.84), 29% (HR = 0.71, 95% CI = 0.65–0.78), 35% (HR = 0.65, 95% CI = 0.58–0.72), 39% (HR = 0.61, 95% CI = 0.54–0.70), 43% (HR = 0.57, 95% CI = 0.49–0.67), and 44% (HR = 0.56, 95% CI = 0.47–0.67) decreased chance of all-cause mortality, respectively. Cancer survivors with a high PA intensity had additional survival benefits (*p* for trend < 0.001). A consistent result on CVD mortality was also detected among the different PA categories (*p* for trend = 0.01), but cancer-specific mortality was not found (*p* for trend = 0.06). After excluding 1285 cancer survivors who died within 24 months of follow up, the results remained robust. As shown in Table S2, the sensitivity analyses of HRs were consistent with the main results, with 0.78 (0.68 to 0.89), 0.76 (0.70 to 0.83), 0.73 (0.66 to 0.80), 0.70 (0.63 to 0.79), 0.60 (0.52 to 0.69), and 0.62 (0.53 to 0.73) for all-cause mortality among cancer survivors who took part in 10 min–1 h, 1–2.5 h, 2.5–5 h, 5–7.5 h, 7.5–13.3 h, and 13.3–24 h of PA, respectively. In addition, PA was inversely associated with the risk of cancer-specific mortality (*p* for trend = 0.033) and CVD mortality (*p* for trend = 0.014). Finally, to evaluate the influence of unmeasured confounding factors, e-values were calculated based on mortality rates from all-cause, cancer, and CVD. E-values (and lower limits of 95% CI) of PA and all-cause, cancer-specific, and CVD mortality were 1.23 (1.2), 1.36 (1.13), 1.23 (1.13), respectively (Figure 4). According to the conclusion, there was an unmeasured variable associated with both PA and all-cause mortality, cancer-specific mortality, and CVD mortality by HRs of 1.23-fold, 1.36-fold, and 1.23-fold, respectively; however, it is unlikely that weaker confounding would affect this association. Therefore, as a result of the E-value and sensitivity analysis, the results were demonstrated to be robust.

## 4. Discussion

Using a nationally large representative population of US adult cancer survivors, the study showed that individuals who participated in leisure-time PA had higher survival benefits than physically inactive cancer survivors. Cancer survivors with high PA intensity can experience additional survival benefits. These relationships were independent of age, sex, marital status, coronary heart disease, race, diabetes, BMI, smoking, alcohol consumption, hypertension, angina, stroke, myocardial infarction, and asthma. Moreover, these conclusions remained stable in stratification and sensitivity analyses. These conclusions provide evidence that cancer survivors who are physically inactive should be encouraged to perform leisure-time PA to improve survival.

To the best of our knowledge, this is the first prospective study to examine the association between PA and all-cause mortality and to reveal a nonlinear relationship between PA per week and all-cause mortality risk in a large nationally representative adult population of cancer survivors. An individual is defined as a cancer survivor from the initial diagnosis of a tumor to the ultimate end of his/her life without considering any cause of death [20]. The number of cancer survivors is rapidly expanding worldwide. A report estimated that, in the US, nearly 16.9 million adult individuals are presently living with malignancy and about 1.6 million new individuals are confirmed every year [21,22]. Furthermore, the population of cancer survivors is predicted to expand to 22.1 million by 2030 in the US [22]. PA after cancer diagnosis has been frequently associated with improvements in several cancer-related outcomes, including physical functioning, quality of life, fatigue, and overall prognosis among breast, colorectal, and prostate cancer survivors [23,24]. Historically, clinicians have suggested that individuals with cancer rest and avoid PA, while previous studies in the 1990s and the 2000s changed this view. PA is usually safe and well-tolerated during and after cancer treatment in breast and prostate cancer survivors and can improve multiple health outcomes [25]. Some existing clinical guidelines recommend proper PA for individuals diagnosed with cancer [23,26,27]. However, the levels of PA are usually low among cancer survivors, and a few cancer survivors follow the present PA guidelines (at least 2.5 h/week of moderate aerobic activity and ≥2 days/week strength exercise) [28]. Thus, further evaluation of the relationship between PA intensity and the risk of death among cancer survivors is required.

Evidence from prospective population-based cancer cohort studies illustrates that PA intensity declines postdiagnosis [29,30]. Low relative intensity of PA, such as around 10 min–1 h/week, may be easy to accomplish by the majority of adult cancer survivors; therefore, it is necessary to identify whether such intensity PA for cancer survivors is helpful. The main results demonstrated that PA for 10 min–1 h/week still had a protective effect on all-cause mortality. We also found a nonlinear association between PA and probability of all-cause mortality. Threshold effect analysis demonstrated that cancer survivors should perform PA for at least 1 h/week, and performing more PA could provide additional survival benefits for cancer survivors. This result implies that even short-term PA attenuates the excess mortality risk in inactive cancer survivors. Furthermore, regular PA intensity still benefits individuals with cancer. Thus, cancer survivors were recommended to “avoid inactivity” and be as physically active as possible. On the other hand, in our study, high-intensity PA for 13.3–24 h/week also showed a protective effect on all-cause mortality. However, the relationship between high-intensity PA and mortality risk in the general population is inconsistent. Previous studies demonstrated an increased mortality risk among very high PA intensities, whereas other studies suggested a beneficial effect [1,31,32]. This inconsistency may be due to the methodological limitations. The basic clinical information between the two groups was not balanced, and the conclusions might be misleading [31]. Another limitation is the relatively small sample size of the subgroup (*n* = 36) [32]. Furthermore, a recent study using the same database with a large adult population to explore the effects of high-intensity PA on mortality risk supported our findings of a protective effect of this PA modality [2]. Nevertheless, their conclusions were drawn from the general population, and thus are not generalizable to cancer survivors. Our results address a key knowledge gap regarding the relationship between PA and mortality risk in cancer survivors. This study presents direct evidence for a link between PA and increased survival after cancer diagnosis. Specifically, this analysis was performed using a nationally representative large population of adult US cancer survivors that included a number of tumor subtypes that showed favorable outcomes in those with better 5-year survival rates (such as breast, prostate, and colorectal cancer) and also in those with lower survival rates (such as ovarian, liver, and pancreatic cancer) [33].

Some behavioral and biological clinical pathways may account for such relationships. First, PA has a direct effect on cancer growth and metastasis. PA has been demonstrated to reduce cancer growth and progression in preclinical research by inducing antineoplastic effects at the systemic and intratumoral levels [34,35,36]. Second, PA contributes to enhanced treatment completion rates and improves the efficacy of cancer treatment. Previous clinical reports have shown that PA reduces certain tumor treatment-related side-effects [23], which may also enhance tumor treatment completion rates [37]. Furthermore, PA may promote tumor treatment efficacy in an additive, sensitizing, or synergistic manner [38]. Third, the majority of cancer survivors experience dramatic life changes that greatly disturb their physical and psychological health. These conditions can result in an inactive lifestyle, leading to premature death [39]. Furthermore, previous experimental reports have demonstrated that an inactive lifestyle is associated with impaired glucose metabolism and elevated systemic inflammation [40], whereas these associations can be weakened by PA [41]. This is in line with the finding of the protective effects of PA on all-cause mortality in cancer survivors.

Our study had several strengths. First, this was a prospective, national cohort study that used a nationally representative sample of cancer survivors in the US, which enabled the findings to be generalized among various cancer survivors. Second, to decrease the influence of reverse causality, we excluded individuals who died within 24 months of follow up in sensitivity analyses and further excluded participants with skin cancer to confirm our findings. Third, multiple possible confounding factors (personal, preexisting illness, and lifestyle) were available at each survey cycle, which were adjusted for in different models. However, our study had certain limitations. First, the measurement of PA was performed using survey responses at a single point in time, which may yield recall bias. The behavioral changes that occur during follow up may not be fully revealed by PA at baseline. Second, owing to the limitations of the NHIS database design, cancer-specific covariates, such as cancer stage, were not included in the NHIS database. Third, although it was not possible to exclude unmeasured confounding factors, we obtained stable results within different subgroups, and sensitivity analyses and e-values also supported the robustness of the conclusion. Thus, it would be helpful to conduct more prospective studies to determine how PA is related to mortality risk across cancer subtypes.

## 5. Conclusions

A study using a nationwide representative sample of US adults showed that leisure-time PA was protective against all-cause mortality among cancer survivors. The risk of death from all causes was nonlinearly correlated with total PA. Threshold effect analysis demonstrated that cancer survivors should perform PA for at least 1 h/week, and that performing more PA could provide additional survival benefits for cancer survivors. Inactive cancer survivors should be encouraged to perform more PA to reduce the risk of all-cause mortality.

## Figures and Tables

**Table 1:** Baseline characteristics according to total leisure time physical activity level, National Health Interview Survey, 1997–2009.

	Leisure Time PA Level, Minutes/Week						
	0	10 min–1 h	1–2.5 h	2.5–5 h	5–7.5 h	7.5–13.3 h	13.3–24 h
Sex, %							
Female	5718 (64.71%)	764 (67.37%)	1959 (65.52%)	1661 (60.84%)	1076 (60.35%)	869 (53.74%)	493 (49.45%)
Male	3119 (35.29%)	370 (32.63%)	1031 (34.48%)	1069 (39.16%)	707 (39.65%)	748 (46.26%)	504 (50.55%)
Age, years, %							
<40	690 (7.81%)	111 (9.79%)	281 (9.40%)	278 (10.18%)	240 (13.46%)	259 (16.02%)	158 (15.85%)
40–59	2227 (25.20%)	388 (34.22%)	928 (31.04%)	928 (33.99%)	629 (35.28%)	620 (38.34%)	328 (32.90%)
≥60	5920 (66.99%)	635 (56.00%)	1781 (59.57%)	1524 (55.82%)	914 (51.26%)	738 (45.64%)	511 (51.25%)
Body mass index, kg/m^2^, %							
<18.5	267 (3.12%)	23 (2.10%)	68 (2.33%)	41 (1.53%)	25 (1.42%)	22 (1.38%)	16 (1.63%)
18.5–24.9	2997 (35.00%)	349 (31.84%)	1083 (37.04%)	1078 (40.34%)	744 (42.27%)	671 (42.15%)	408 (41.68%)
25–29.9	2983 (34.83%)	385 (35.13%)	1068 (36.53%)	1031 (38.59%)	650 (36.93%)	621 (39.01%)	362 (36.98%)
≥30	2317 (27.06%)	339 (30.93%)	705 (24.11%)	522 (19.54%)	341 (19.38%)	278 (17.46%)	193 (19.71%)
Race/ethnicity, %							
White	7645 (86.51%)	979 (86.33%)	2706 (90.50%)	2503 (91.68%)	1640 (91.98%)	1495 (92.46%)	900 (90.27%)
Black	957 (10.83%)	118 (10.41%)	201 (6.72%)	168 (6.15%)	87 (4.88%)	79 (4.89%)	60 (6.02%)
Other	235 (2.66%)	37 (3.26%)	83 (2.78%)	59 (2.16%)	56 (3.14%)	43 (2.66%)	37 (3.71%)
Marital status, %							
Married/Living with partner	4074 (46.18%)	582 (51.41%)	1592 (53.30%)	1539 (56.46%)	1028 (57.69%)	975 (60.30%)	572 (57.49%)
Divorced/separated/widowed	4062 (46.04%)	453 (40.02%)	1152 (38.57%)	923 (33.86%)	578 (32.44%)	472 (29.19%)	324 (32.56%)
Never married	686 (7.78%)	97 (8.57%)	243 (8.14%)	264 (9.68%)	176 (9.88%)	170 (10.51%)	99 (9.95%)
Alcohol drinking, %							
Lifetime abstainer	2630 (37.20%)	213 (26.30%)	550 (25.87%)	427 (22.71%)	232 (19.46%)	174 (16.84%)	145 (21.71%)
Former drinker	1864 (26.37%)	206 (25.43%)	512 (24.08%)	432 (22.98%)	252 (21.14%)	194 (18.78%)	150 (22.46%)
Current drinker	2575 (36.43%)	391 (48.27%)	1064 (50.05%)	1021 (54.31%)	708 (59.40%)	665 (64.38%)	373 (55.84%)
Smoking status, %							
Never	1806 (20.50%)	191 (16.84%)	464 (15.54%)	357 (13.09%)	269 (15.10%)	211 (13.07%)	162 (16.27%)
Former	1750 (19.86%)	209 (18.43%)	607 (20.34%)	592 (21.71%)	368 (20.65%)	358 (22.18%)	214 (21.49%)
Current	3510 (39.84%)	472 (41.62%)	1241 (41.57%)	1133 (41.55%)	748 (41.98%)	667 (41.33%)	399 (40.06%)
Smoker, current status unknown	1745 (19.80%)	262 (23.10%)	673 (22.55%)	645 (23.65%)	397 (22.28%)	378 (23.42%)	221 (22.19%)
Diabetes, %							
No	7228 (81.83%)	969 (85.45%)	2578 (86.28%)	2377 (87.07%)	1574 (88.28%)	1468 (90.79%)	904 (90.67%)
Yes	1462 (16.55%)	150 (13.23%)	354 (11.85%)	300 (10.99%)	176 (9.87%)	130 (8.04%)	77 (7.72%)
Borderline	143 (1.62%)	15 (1.32%)	56 (1.87%)	53 (1.94%)	33 (1.85%)	19 (1.18%)	16 (1.60%)
Hypertension, %							
No	4178 (47.32%)	572 (50.49%)	1610 (53.90%)	1579 (57.86%)	1064 (59.74%)	1060 (65.55%)	606 (60.84%)
Yes	4652 (52.68%)	561 (49.51%)	1377 (46.10%)	1150 (42.14%)	717 (40.26%)	557 (34.45%)	390 (39.16%)
Coronary heart disease, %							
No	7763 (88.10%)	1026 (90.72%)	2711 (90.97%)	2485 (91.16%)	1640 (92.24%)	1501 (92.88%)	916 (92.06%)
Yes	1049 (11.90%)	105 (9.28%)	269 (9.03%)	241 (8.84%)	138 (7.76%)	115 (7.12%)	79 (7.94%)
Angina, %							
No	8145 (92.41%)	1070 (94.52%)	2793 (93.69%)	2590 (94.98%)	1691 (95.05%)	1543 (95.48%)	959 (96.29%)
Yes	669 (7.59%)	62 (5.48%)	188 (6.31%)	137 (5.02%)	88 (4.95%)	73 (4.52%)	37 (3.71%)
Myocardial infarction, %							
No	7879 (89.34%)	1048 (92.50%)	2778 (93.07%)	2544 (93.29%)	1688 (94.78%)	1523 (94.25%)	945 (94.78%)
Yes	940 (10.66%)	85 (7.50%)	207 (6.93%)	183 (6.71%)	93 (5.22%)	93 (5.75%)	52 (5.22%)
Stroke, %							
No	8036 (91.12%)	1071 (94.69%)	2853 (95.42%)	2621 (96.08%)	1721 (96.74%)	1580 (97.71%)	957 (95.99%)
Yes	783 (8.88%)	60 (5.31%)	137 (4.58%)	107 (3.92%)	58 (3.26%)	37 (2.29%)	40 (4.01%)
Asthma, %							
No	7618 (86.31%)	981 (86.74%)	2623 (87.84%)	2409 (88.34%)	1578 (88.65%)	1425 (88.13%)	889 (89.17%)
Yes	1208 (13.69%)	150 (13.26%)	363 (12.16%)	318 (11.66%)	202 (11.35%)	192 (11.87%)	108 (10.83%)

**Table 2:** Associations of physical activity with all-cause mortality and cancer mortality among cancer survivors.

	Non-Adjusted	Adjust I	Adjust II
All-cause mortality			
PA(Continuous)	0.92 (0.91, 0.92) < 0.0001	0.94 (0.93, 0.95) < 0.0001	0.95 (0.95, 0.96) < 0.0001
PA level			
0	1 (Reference)	1 (Reference)	1 (Reference)
10 min–1 h	0.60 (0.54, 0.67) < 0.0001	0.71 (0.64, 0.79) < 0.0001	0.75 (0.67, 0.85) < 0.0001
1–2.5 h	0.60 (0.56, 0.64) < 0.0001	0.67 (0.63, 0.72) < 0.0001	0.72 (0.67, 0.78) < 0.0001
2.5–5 h	0.48 (0.45, 0.52) < 0.0001	0.58 (0.54, 0.63) < 0.0001	0.66 (0.60, 0.72) < 0.0001
5–7.5 h	0.43 (0.39, 0.48) < 0.0001	0.55 (0.50, 0.61) < 0.0001	0.63 (0.56, 0.70) < 0.0001
7.5–13.3 h	0.35 (0.32, 0.40) < 0.0001	0.48 (0.42, 0.53) < 0.0001	0.53 (0.47, 0.61) < 0.0001
13.3–24 h	0.39 (0.34, 0.45) < 0.0001	0.50 (0.43, 0.57) < 0.0001	0.57 (0.49, 0.66) < 0.0001
*p* for trend	<0.001	<0.001	<0.001
Cancer mortality			
PA(Continuous)	0.89 (0.83, 0.96) 0.0027	0.91 (0.85, 0.98) 0.0114	0.90 (0.83, 0.98) 0.0115
PA level			
0	1 (Reference)	1 (Reference)	1 (Reference)
10 min–1 h	0.64 (0.26, 1.58) 0.3328	0.70 (0.28, 1.73) 0.4362	0.86 (0.35, 2.15) 0.7535
1–2.5 h	0.25 (0.11, 0.58) 0.0011	0.28 (0.12, 0.64) 0.0025	0.23 (0.08, 0.64) 0.0046
2.5–5 h	0.48 (0.24, 0.96) 0.0371	0.55 (0.27, 1.10) 0.0884	0.61 (0.29, 1.30) 0.2007
5–7.5 h	0.31 (0.11, 0.84) 0.0212	0.36 (0.13, 0.98) 0.0461	0.42 (0.15, 1.17) 0.0986
7.5–13.3 h	0.29 (0.09, 0.93) 0.0365	0.34 (0.11, 1.09) 0.0701	0.29 (0.07, 1.20) 0.0872
13.3–24 h	0.45 (0.14, 1.43) 0.1771	0.48 (0.15, 1.55) 0.2208	0.37 (0.09, 1.51) 0.1646
*p* for trend	0.003	0.011	0.011
Cardiovascular disease mortality			
PA(Continuous)	0.96 (0.93, 0.99) 0.0084	0.96 (0.93, 0.99) 0.0083	0.95 (0.91, 0.98) 0.0052
PA level			
0	1 (Reference)	1 (Reference)	1 (Reference)
10 min–1 h	1.24 (0.79, 1.92) 0.3478	1.32 (0.85, 2.05) 0.2222	1.39 (0.88, 2.20) 0.1632
1–2.5 h	0.83 (0.60, 1.14) 0.2530	0.88 (0.63, 1.22) 0.4368	0.84 (0.59, 1.22) 0.3674
2.5–5 h	0.80 (0.55, 1.15) 0.2232	0.84 (0.58, 1.22) 0.3643	0.69 (0.45, 1.06) 0.0906
5–7.5 h	0.89 (0.59, 1.34) 0.5828	0.90 (0.60, 1.36) 0.6229	0.87 (0.55, 1.37) 0.5429
7.5–13.3 h	0.68 (0.41, 1.14) 0.1412	0.71 (0.43, 1.19) 0.1979	0.60 (0.32, 1.11) 0.1027
13.3–24 h	0.46 (0.22, 0.98) 0.0447	0.40 (0.18, 0.91) 0.0294	0.40 (0.16, 0.99) 0.0470
*p* for trend	0.008	0.003	0.005

Non-adjusted model adjusts for: None; Adjust I model adjust for: age, sex, BMI, race, marital status, smoking, alcohol drinking; Adjust II model adjust for: age, sex, BMI, race, marital status, smoking, alcohol drinking, hypertension, coronary heart disease, angina, myocardial infarction, stroke, diabetes, and asthma.

**Table 3:** Threshold effect analysis of physical activity on all-cause mortality using piecewise binary logistic regression models.

	Inflection Point	Group	HR (95% CI)	*p* Value	*p* for Log Likelihood Ratio Test
Physical activity	1 h/week	≤1	0.70 (0.66, 0.75)	<0.001	<0.001
		>1	0.98 (0.97, 0.99)	<0.001	

**Table 4:** Subgroup analyses for the associations between physical activity and all-cause mortality among cancer survivors.

Variable	Participants, No.	0	10 min–1 h	1–2.5 h	2.5–5 h	5–7.5 h	7.5–13.3 h	13.3–24 h	*p* Value for Interaction
Age, y									0.7287
<40	2017	1 [Reference]	0.77 (0.33, 1.83) 0.5598	0.78 (0.44, 1.40) 0.4113	0.40 (0.19, 0.84) 0.0155	0.64 (0.32, 1.28) 0.2076	0.61 (0.31, 1.21) 0.1573	0.51 (0.20, 1.29) 0.1561	
40–59	6048	1 [Reference]	0.88 (0.65, 1.19) 0.4071	0.70 (0.56, 0.89) 0.0029	0.57 (0.44, 0.73) < 0.0001	0.66 (0.50, 0.88) 0.0041	0.53 (0.38, 0.73) < 0.0001	0.45 (0.29, 0.68) 0.0002	
≥60	12023	1 [Reference]	0.70 (0.61, 0.80) < 0.0001	0.68 (0.62, 0.74) < 0.0001	0.59 (0.54, 0.65) < 0.0001	0.56 (0.49, 0.63) < 0.0001	0.44 (0.38, 0.51) < 0.0001	0.50 (0.42, 0.59) < 0.0001	
Sex									0.603
Female	12540	1 [Reference]	0.77 (0.66, 0.91) 0.0016	0.70 (0.63, 0.78) < 0.0001	0.66 (0.58, 0.75) < 0.0001	0.64 (0.55, 0.75) < 0.0001	0.52 (0.42, 0.64) < 0.0001	0.49 (0.38, 0.64) < 0.0001	
Male	7548	1 [Reference]	0.72 (0.60, 0.87) 0.0008	0.76 (0.67, 0.86) < 0.0001	0.65 (0.57, 0.74) < 0.0001	0.62 (0.53, 0.73) < 0.0001	0.54 (0.45, 0.64) < 0.0001	0.62 (0.51, 0.74) < 0.0001	
BMI, Kg/m^2^									0.1238
<18.5	462	1 [Reference]	0.76 (0.32, 1.80) 0.5259	0.69 (0.44, 1.07) 0.0951	0.42 (0.23, 0.79) 0.0068	0.33 (0.14, 0.80) 0.0140	0.44 (0.13, 1.47) 0.1815	0.53 (0.16, 1.71) 0.2851	
18.5–24.9	7330	1 [Reference]	0.76 (0.32, 1.80) 0.5259	0.69 (0.44, 1.07) 0.0951	0.42 (0.23, 0.79) 0.0068	0.33 (0.14, 0.80) 0.0140	0.44 (0.13, 1.47) 0.1815	0.53 (0.16, 1.71) 0.2851	
25–29.9	7100	1 [Reference]	0.73 (0.59, 0.90) 0.0034	0.78 (0.68, 0.89) 0.0002	0.71 (0.62, 0.82) < 0.0001	0.74 (0.62, 0.89) 0.0012	0.62 (0.50, 0.75) < 0.0001	0.57 (0.44, 0.74) < 0.0001	
≥30	4695	1 [Reference]	0.82 (0.64, 1.04) 0.0937	0.63 (0.53, 0.77) < 0.0001	0.65 (0.52, 0.80) < 0.0001	0.50 (0.38, 0.67) < 0.0001	0.56 (0.41, 0.77) 0.0004	0.82 (0.60, 1.12) 0.2066	
Race/ethnicity									0.5269
White	17868	1 [Reference]	0.75 (0.66, 0.86) < 0.0001	0.72 (0.66, 0.78) < 0.0001	0.67 (0.61, 0.73) < 0.0001	0.65 (0.58, 0.73) < 0.0001	0.54 (0.47, 0.62) < 0.0001	0.57 (0.49, 0.67) < 0.0001	
Black	1670	1 [Reference]	0.69 (0.46, 1.02) 0.0640	0.72 (0.54, 0.96) 0.0229	0.54 (0.38, 0.77) 0.0006	0.55 (0.35, 0.86) 0.0093	0.59 (0.33, 1.02) 0.0606	0.80 (0.47, 1.38) 0.4282	
Other	550	1 [Reference]	1.12 (0.59, 2.12) 0.7376	0.61 (0.30, 1.27) 0.1906	0.80 (0.42, 1.53) 0.5025	0.37 (0.15, 0.90) 0.0287	0.32 (0.11, 0.93) 0.0360	0.16 (0.02, 1.15) 0.0678	
Marital status									0.907
Married/Living with partner	10362	1 [Reference]	0.75 (0.62, 0.90) 0.0018	0.69 (0.61, 0.78) < 0.0001	0.67 (0.59, 0.76) < 0.0001	0.62 (0.53, 0.73) < 0.0001	0.54 (0.45, 0.65) < 0.0001	0.55 (0.44, 0.68) < 0.0001	
Divorced/separated/widowed	7964	1 [Reference]	0.73 (0.61, 0.87) 0.0004	0.74 (0.67, 0.83) < 0.0001	0.64 (0.56, 0.73) < 0.0001	0.66 (0.56, 0.78) < 0.0001	0.51 (0.41, 0.63) < 0.0001	0.58 (0.46, 0.73) < 0.0001	
Never married	1735	1 [Reference]	1.11 (0.69, 1.77) 0.6709	0.72 (0.51, 1.01) 0.0605	0.67 (0.45, 0.98) 0.0403	0.52 (0.32, 0.85) 0.0082	0.68 (0.42, 1.11) 0.1247	0.62 (0.34, 1.13) 0.1195	
Alcohol drinking									0.941
Lifetime abstainer	4371	1 [Reference]	0.80 (0.64, 1.00) 0.0540	0.70 (0.60, 0.82) < 0.0001	0.68 (0.57, 0.82) < 0.0001	0.60 (0.47, 0.76) < 0.0001	0.52 (0.38, 0.71) < 0.0001	0.61 (0.45, 0.84) 0.0025	
Former drinker	3610	1 [Reference]	0.78 (0.63, 0.96) 0.0218	0.69 (0.60, 0.80) < 0.0001	0.60 (0.51, 0.71) < 0.0001	0.61 (0.49, 0.75) < 0.0001	0.48 (0.36, 0.64) < 0.0001	0.62 (0.47, 0.82) 0.0007	
Current drinker	6797	1 [Reference]	0.71 (0.59, 0.86) 0.0006	0.75 (0.66, 0.85) < 0.0001	0.69 (0.61, 0.79) < 0.0001	0.67 (0.57, 0.78) < 0.0001	0.57 (0.48, 0.68) < 0.0001	0.54 (0.43, 0.67) < 0.0001	
Smoking status									0.032
Never	3460	1 [Reference]	0.99 (0.74, 1.34) 0.9635	0.74 (0.60, 0.92) 0.0054	0.54 (0.41, 0.71) < 0.0001	0.78 (0.60, 1.03) 0.0807	0.80 (0.59, 1.09) 0.1512	0.60 (0.41, 0.88) 0.0079	
Former	4089	1 [Reference]	0.74 (0.60, 0.92) 0.0066	0.73 (0.64, 0.83) < 0.0001	0.69 (0.60, 0.80) < 0.0001	0.70 (0.59, 0.85) 0.0002	0.54 (0.44, 0.66) < 0.0001	0.60 (0.48, 0.76) < 0.0001	
Current	8170	1 [Reference]	0.64 (0.52, 0.77) < 0.0001	0.68 (0.60, 0.77) < 0.0001	0.64 (0.56, 0.74) < 0.0001	0.54 (0.45, 0.65) < 0.0001	0.44 (0.35, 0.55) < 0.0001	0.50 (0.39, 0.65) < 0.0001	
Diabetes									0.9089
No	17098	1 [Reference]	0.74 (0.65, 0.85) < 0.0001	0.72 (0.66, 0.79) < 0.0001	0.65 (0.59, 0.72) < 0.0001	0.63 (0.55, 0.71) < 0.0001	0.50 (0.43, 0.58) < 0.0001	0.55 (0.47, 0.65) < 0.0001	
Yes	2649	1 [Reference]	0.80 (0.60, 1.05) 0.1086	0.68 (0.56, 0.83) 0.0002	0.67 (0.54, 0.82) 0.0002	0.67 (0.51, 0.88) 0.0041	0.70 (0.52, 0.95) 0.0215	0.77 (0.51, 1.15) 0.1979	
Hypertension									0.0435
No	10669	1 [Reference]	0.69 (0.57, 0.84) 0.0002	0.77 (0.68, 0.86) < 0.0001	0.65 (0.57, 0.74) < 0.0001	0.59 (0.50, 0.70) < 0.0001	0.47 (0.39, 0.57) < 0.0001	0.49 (0.39, 0.62) < 0.0001	
Yes	9404	1 [Reference]	0.80 (0.68, 0.93) 0.0046	0.68 (0.61, 0.76) < 0.0001	0.66 (0.58, 0.74) < 0.0001	0.66 (0.57, 0.77) < 0.0001	0.60 (0.50, 0.72) < 0.0001	0.64 (0.53, 0.79) < 0.0001	
Coronary heart disease									0.0428
No	18042	1 [Reference]	0.75 (0.66, 0.86) < 0.0001	0.73 (0.67, 0.79) < 0.0001	0.63 (0.57, 0.69) < 0.0001	0.61 (0.54, 0.69) < 0.0001	0.53 (0.46, 0.61) < 0.0001	0.53 (0.44, 0.62) < 0.0001	
Yes	1996	1 [Reference]	0.76 (0.55, 1.05) 0.0944	0.70 (0.56, 0.86) 0.0007	0.81 (0.65, 1.01) 0.0560	0.77 (0.58, 1.02) 0.0661	0.53 (0.37, 0.75) 0.0003	0.88 (0.62, 1.25) 0.4626	
Angina									0.2168
No	18791	1 [Reference]	0.76 (0.67, 0.86) < 0.0001	0.72 (0.66, 0.78) < 0.0001	0.64 (0.58, 0.70) < 0.0001	0.60 (0.54, 0.68) < 0.0001	0.54 (0.47, 0.62) < 0.0001	0.57 (0.49, 0.67) < 0.0001	
Yes	1254	1 [Reference]	0.67 (0.45, 1.01) 0.0585	0.72 (0.57, 0.92) 0.0081	0.80 (0.61, 1.04) 0.0992	0.93 (0.66, 1.30) 0.6648	0.50 (0.33, 0.76) 0.0013	0.51 (0.28, 0.92) 0.0258	
Myocardial infarction									0.3738
No	18405	1 [Reference]	0.77 (0.67, 0.87) < 0.0001	0.72 (0.66, 0.78) < 0.0001	0.63 (0.58, 0.70) < 0.0001	0.62 (0.55, 0.70) < 0.0001	0.54 (0.47, 0.61) < 0.0001	0.57 (0.48, 0.67) < 0.0001	
Yes	1653	1 [Reference]	0.62 (0.44, 0.88) 0.0066	0.69 (0.55, 0.86) 0.0012	0.80 (0.63, 1.01) 0.0571	0.66 (0.47, 0.92) 0.0149	0.49 (0.32, 0.73) 0.0005	0.57 (0.35, 0.92) 0.0218	
Stroke									0.0876
No	18839	1 [Reference]	0.79 (0.70, 0.90) 0.0003	0.73 (0.67, 0.79) < 0.0001	0.66 (0.60, 0.72) < 0.0001	0.63 (0.56, 0.70) < 0.0001	0.53 (0.46, 0.61) < 0.0001	0.55 (0.47, 0.65) < 0.0001	
Yes	1222	1 [Reference]	0.43 (0.27, 0.69) 0.0004	0.66 (0.51, 0.86) 0.0018	0.66 (0.48, 0.91) 0.0102	0.62 (0.41, 0.95) 0.0264	0.50 (0.27, 0.92) 0.0249	0.95 (0.56, 1.61) 0.8575	
Asthma									0.7195
No	17523	1 [Reference]	0.76 (0.67, 0.87) < 0.0001	0.72 (0.66, 0.79) < 0.0001	0.66 (0.61, 0.73) < 0.0001	0.63 (0.56, 0.70) < 0.0001	0.54 (0.47, 0.62) < 0.0001	0.56 (0.48, 0.65) < 0.0001	
Yes	2541	1 [Reference]	0.74 (0.52, 1.04) 0.0853	0.71 (0.56, 0.91) 0.0073	0.61 (0.46, 0.81) 0.0006	0.64 (0.44, 0.94) 0.0241	0.44 (0.27, 0.71) 0.0009	0.78 (0.44, 1.36) 0.3772	

## Data Availability

Publicly available data were used in this study. The raw data used are available from https://www.cdc.gov/nchs/nhis/index.htm (accessed on 30 November 2021).

## Supplementary Materials

The following supporting information can be downloaded at: https://www.mdpi.com/article/10.3390/cancers14235760/s1, Figure S1: Distribution histogram of physical activity level (minutes/week); Figure S2: The number of cancer survivors in 13 survey cycles; Figure S3: Detailed number of cancer survivors included in this study; Table S1: Sensitivity analyses of the associations between physical activities and all-cause mortality among cancer survivors excluding those with skin cancer; Table S2: Sensitivity analyses by excluding those who died in the first 2 years of follow-up.
