# Supplementary material for: Association of Physical Activity Intensity with All-Cause Mortality in Cancer Survivors: A National Prospective Cohort Study
Source: Cancers (Basel). 2022 Nov 23;14(23):5760. doi: 10.3390/cancers14235760 (PMC9740265; doi:10.3390/cancers14235760)
Supplement: Supplementary file 1 [file cancers-14-05760-s001.zip › cancers-1945219-supplementary.pdf]

cancers

MDPI

Supplementary materials

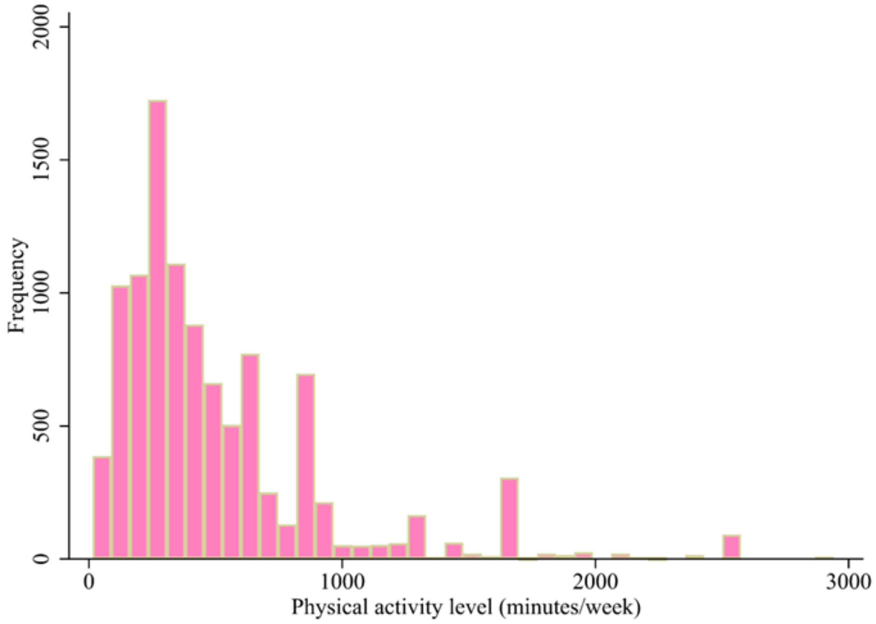

Figure S1. Distribution histogram of physical activity level (minutes/week).

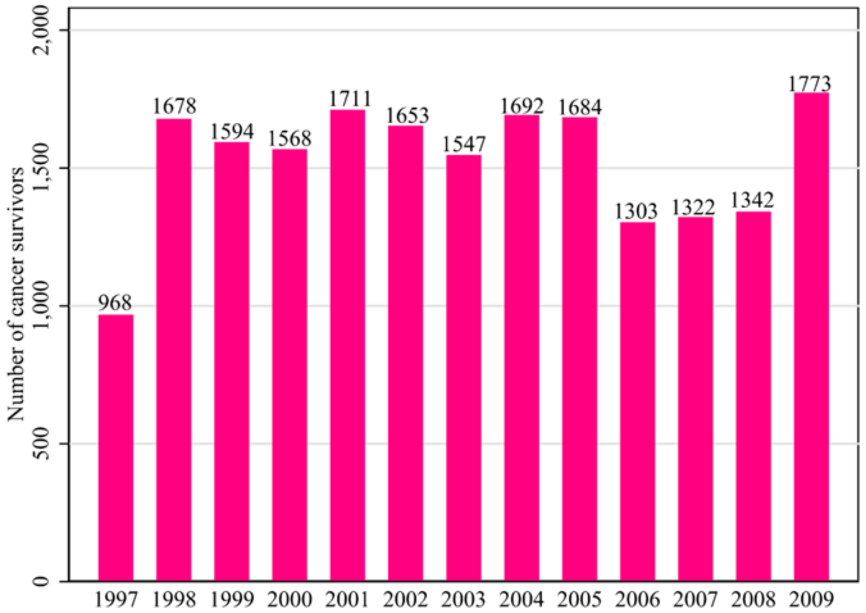

Figure S2. The number of cancer survivors in 13 survey cycles.

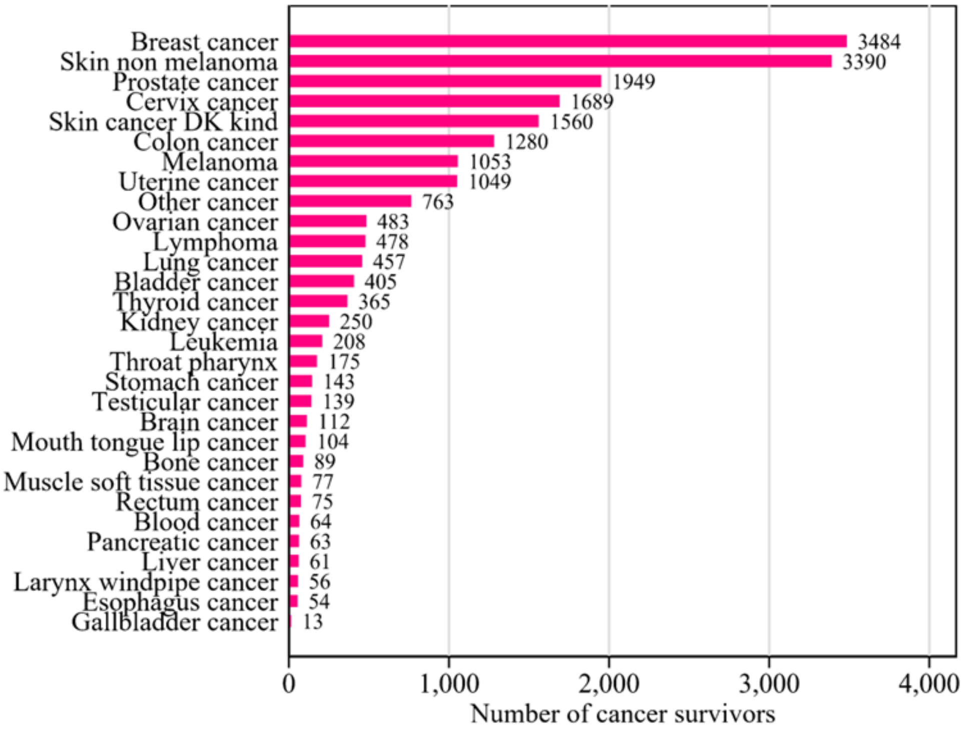

Figure S3. Detailed number of cancer survivors included in this study.

Table S1. Sensitivity analyses of the associations between physical activities and all-cause mortality among cancer survivors excluding those with skin cancer.

	Non-adjusted	Adjust I	Adjust II
All-cause mortality			
PA(Continuous)	0.92 (0.91, 0.93) <0.0001	0.95 (0.94, 0.96) <0.0001	0.95 (0.94, 0.96) <0.0001
PA level			
0	1(Reference)	1(Reference)	1(Reference)
10min-1h	0.59 (0.52, 0.67) <0.0001	0.72 (0.63, 0.83) <0.0001	0.73 (0.63, 0.84) <0.0001
1-2.5h	0.61 (0.56, 0.66) <0.0001	0.69 (0.63, 0.76) <0.0001	0.71 (0.65, 0.78) <0.0001
2.5-5h	0.49 (0.45, 0.54) <0.0001	0.64 (0.57, 0.71) <0.0001	0.65 (0.58, 0.72) <0.0001
5-7.5h	0.43 (0.38, 0.48) <0.0001	0.59 (0.52, 0.68) <0.0001	0.61 (0.54, 0.70) <0.0001
7.5-13.3h	0.39 (0.34, 0.45) <0.0001	0.55 (0.47, 0.65) <0.0001	0.57 (0.49, 0.67) <0.0001
13.3-24h	0.41 (0.35, 0.49) <0.0001	0.53 (0.44, 0.63) <0.0001	0.56 (0.47, 0.67) <0.0001
P for trend	<0.001	<0.001	<0.001
Cancer mortality			
PA(Continuous)	0.92 (0.85, 1.00) 0.0472	0.92 (0.83, 1.01) 0.0708	0.91 (0.83, 1.00) 0.0595
PA level			
0	1(Reference)	1(Reference)	1(Reference)
10min-1h	0.60 (0.19, 1.92) 0.3912	0.78 (0.24, 2.52) 0.6823	0.86 (0.27, 2.76) 0.7938
1-2.5h	0.32 (0.13, 0.79) 0.0141	0.35 (0.13, 0.98) 0.0465	0.37 (0.13, 1.04) 0.0589
2.5-5h	0.68 (0.32, 1.42) 0.3004	0.85 (0.38, 1.90) 0.6928	0.83 (0.37, 1.88) 0.6631
5-7.5h	0.25 (0.06, 1.03) 0.0543	0.35 (0.09, 1.47) 0.1522	0.36 (0.09, 1.52) 0.1661
7.5-13.3h	0.49 (0.15, 1.56) 0.2275	0.52 (0.12, 2.17) 0.3698	0.54 (0.13, 2.26) 0.4017
13.3-24h	0.51 (0.12, 2.08) 0.3467	0.31 (0.04, 2.27) 0.2494	0.26 (0.04, 1.95) 0.1921
P for trend	0.047	0.071	0.06
Cardiovascular disease mortality			
PA(Continuous)	0.94 (0.90, 0.98) 0.0051	0.92 (0.88, 0.97) 0.0030	0.93 (0.89, 0.98) 0.0097
PA level			
0	1(Reference)	1(Reference)	1(Reference)
10min-1h	1.22 (0.72, 2.08) 0.4577	1.37 (0.78, 2.38) 0.2713	1.42 (0.81, 2.49) 0.2180
1-2.5h	0.67 (0.44, 1.02) 0.0609	0.65 (0.41, 1.04) 0.0727	0.65 (0.40, 1.04) 0.0740
2.5-5h	0.80 (0.52, 1.24) 0.3219	0.75 (0.46, 1.23) 0.2573	0.67 (0.41, 1.11) 0.1194
5-7.5h	0.77 (0.45, 1.32) 0.3439	0.74 (0.41, 1.31) 0.2989	0.85 (0.47, 1.51) 0.5736
7.5-13.3h	0.65 (0.34, 1.23) 0.1833	0.58 (0.27, 1.25) 0.1618	0.61 (0.28, 1.33) 0.2153

13.3-24h	0.20 (0.05, 0.81) 0.0236	0.12 (0.02, 0.88) 0.0365	0.15 (0.02, 1.08) 0.0598
P for trend	0.005	0.003	0.01

Non-adjusted model adjust for: None; Adjust I model adjust for: age, sex, BMI, race, marital status, smoking, alcohol drinking; Adjust II model adjust for: age, sex, BMI, race, marital status, smoking, alcohol drinking, hypertension, coronary heart disease, angina, myocardial infarction, stroke, diabetes, and asthma;.

Table S2. Sensitivity analyses by excluding those who died in the first 2 years of follow-up.

	Non-adjusted	Adjust I	Adjust II
All-cause mortality			
PA(Continuous)	0.93 (0.92, 0.94) <0.0001	0.96 (0.95, 0.97) <0.0001	0.96 (0.96, 0.97) <0.0001
PA level			
0	1(Reference)	1(Reference)	1(Reference)
10min-1h	0.62 (0.55, 0.70) <0.0001	0.78 (0.68, 0.89) 0.0002	0.78 (0.68, 0.89) 0.0002
1-2.5h	0.63 (0.58, 0.68) <0.0001	0.75 (0.68, 0.81) <0.0001	0.76 (0.70, 0.83) <0.0001
2.5-5h	0.53 (0.49, 0.57) <0.0001	0.72 (0.65, 0.79) <0.0001	0.73 (0.66, 0.80) <0.0001
5-7.5h	0.48 (0.43, 0.53) <0.0001	0.69 (0.61, 0.77) <0.0001	0.70 (0.63, 0.79) <0.0001
7.5-13.3h	0.39 (0.35, 0.44) <0.0001	0.58 (0.50, 0.66) <0.0001	0.60 (0.52, 0.69) <0.0001
13.3-24h	0.43 (0.37, 0.49) <0.0001	0.59 (0.50, 0.69) <0.0001	0.62 (0.53, 0.73) <0.0001
P for trend	<0.001	<0.001	<0.001
Cancer mortality			
PA(Continuous)	0.91 (0.85, 0.98) 0.0119	0.91 (0.84, 0.99) 0.0297	0.92 (0.84, 0.99) 0.0326
PA level			
0	1(Reference)	1(Reference)	1(Reference)
10min-1h	0.60 (0.22, 1.64) 0.3211	0.76 (0.27, 2.09) 0.5878	0.78 (0.28, 2.16) 0.6356
1-2.5h	0.29 (0.13, 0.67) 0.0038	0.25 (0.09, 0.70) 0.0083	0.26 (0.09, 0.73) 0.0100
2.5-5h	0.56 (0.28, 1.11) 0.0968	0.68 (0.32, 1.45) 0.3182	0.70 (0.33, 1.50) 0.3635
5-7.5h	0.35 (0.13, 0.96) 0.0408	0.47 (0.17, 1.32) 0.1531	0.49 (0.17, 1.35) 0.1669
7.5-13.3h	0.33 (0.10, 1.05) 0.0609	0.31 (0.08, 1.30) 0.1093	0.32 (0.08, 1.32) 0.1151
13.3-24h	0.52 (0.16, 1.64) 0.2608	0.42 (0.10, 1.72) 0.2272	0.42 (0.10, 1.74) 0.2323
P for trend	0.012	0.03	0.033
Cardiovascular disease mortality			
PA(Continuous)	0.97 (0.93, 1.00) 0.0372	0.95 (0.91, 0.99) 0.0121	0.95 (0.91, 0.99) 0.0138
PA level			
0	1(Reference)	1(Reference)	1(Reference)
10min-1h	1.13 (0.68, 1.85) 0.6419	1.28 (0.76, 2.14) 0.3580	1.20 (0.71, 2.03) 0.4904
1-2.5h	0.79 (0.55, 1.12) 0.1871	0.78 (0.52, 1.15) 0.2073	0.78 (0.52, 1.16) 0.2195
2.5-5h	0.79 (0.53, 1.17) 0.2328	0.75 (0.48, 1.18) 0.2115	0.72 (0.46, 1.13) 0.1523
5-7.5h	0.93 (0.61, 1.43) 0.7563	0.84 (0.52, 1.36) 0.4725	0.86 (0.53, 1.40) 0.5504
7.5-13.3h	0.72 (0.42, 1.22) 0.2239	0.59 (0.31, 1.14) 0.1144	0.59 (0.31, 1.13) 0.1128
13.3-24h	0.52 (0.25, 1.12) 0.0947	0.44 (0.18, 1.07) 0.0709	0.44 (0.18, 1.08) 0.0728
P for trend	0.037	0.012	0.014

Non-adjusted model adjust for: None; Adjust I model adjust for: age, sex, BMI, race, marital status, smoking, alcohol drinking; Adjust II model adjust for: age, sex, BMI, race, marital status, smoking, alcohol drinking, hypertension, coronary heart disease, angina, myocardial infarction, stroke, diabetes, and asthma;.
